# Surgery

**Published:** 1824-09-01

**Authors:** 


### Surgery.

1. *Injuries of the Head—Concussion, &c.** The motto of the medical practitioner should be "*nil desperandum.*" We occasionally, we might say *often*, see such extraordinary recoveries or escapes from the very jaws of Cerberus, that we cannot be too cautious in our prognosis, or too careful in persevering both in our visits and remedial agents (however inert) while life remains. We have been led to these reflections, at this moment, by the perusal of Mr. Shoveller's case, in the June number of our respected cotemporary. The patient was a seaman, twenty-seven years of age, short and muscular, who was struck by a block, while aloft, and precipitated on the deck below, from a height of about thirteen feet. He was taken up insensible, with stertorous breathing, slow pulse, dilated pupils, and other symptoms of compression. He was bled to thirty ounces, when he became faint, but opened his eyes,

* Case of severe injury of the brain, &c. By W. Shoveller, Esq. Surgeon of His Majesty's Ship Jupiter.—*Med. Repos.* No. 6, *New Series.*

and uttered some incoherent words, falling back again into a state of insensibility. On examining the head, a tumour, the size of a pigeon's egg, was perceived over the right parietal bone, but no fracture or depression could be distinguished. At four, p. m. of the same day, he was again bled to twenty-four ounces, and had ten grains of calomel; the head to be covered with cold wet cloths. *2nd day*, is still soporose, and appears delirious, with great oppression. The skull laid bare, at the place above-mentioned, exhibited the symptoms of injury. He lost twelve ounces of blood by this operation, and seemed relieved. *Evening.* Delirium with extreme restlessness—pulse 110, with reaction on the surface. Venesection to twenty-four ounces, when faintness supervened. Ten grains of calomel—an enema—head to be kept wet and cold. *3d day.* Has passed a better night—enema has operated well—pulse 84. But he is still delirious. Five grains of calomel, and three of antimonial powder every three hours. *4th day.* Had a bad delirious night—pulse 90 and firm; temperature increased—bled to twenty-four ounces. *5th day.* Still delirious and restless, with febrile excitement. Mouth slightly sore from the mercury;—this omitted; to have digitalis and nitrate of potass three or four times a day. The temporal artery opened, and eight ounces of blood detracted. *6th day.* Delirium and jactitation excessive last night, and nearly the same to day—pulse 90 and firm—tongue white—fever—bowels free. Venesection to twenty ounces, and a blister to the scalp. *7th day.* Has passed the night better—talks incoherently—and has still much jactitation—pulse 86—skin cool. In the evening there were strong symptoms of debility, and some sago and wine was ordered. *8th day.* Seems rather better—pulse 84—less mental aberration. *9th day.* Continues to improve. In the evening a relapse. Delirium, flushing of the face—violent throbbing of the left temporal artery—pulse 100 and firm. Temporal arteriotomy to twelve ounces, after which he became more calm. Bowels free. *10th day.* His mouth is more affected by mercury—but there is no regular ptyalism—had a bad delirious night—passes his stools involuntarily. To have wine and sago every two hours, with decoctum cinchonæ. 11th day. "Countenance assumes a more morbid appearance, and is indicative of sinking. Low muttering delirium—pulse feeble—Alvine discharges are still frequent, and passed in his bed." In this almost hopeless condition he remained five days, soiling and destroying all his bed-clothes by involuntary excretions. By this time the integuments over the sacrum, &c. were ulcerated. From this period, however, he gradually, though slowly recovered—at least to a certain extent. He has numbness and feeble power of the lower extremities—indistinct articulation—tongue, when projected, is tremulous, and drawn to the left side—his recollection, especially as to the Jupiter, in which he was actually embarked, has totally failed him. Nine months after the accident, his intellects were almost entirely restored—but his lower extremities are still tremulous and paralytic—his general health and appearance improving. Invalided.

We think it will be admitted that the above patient was in a very hopeless condition for some time, and that most surgeons, young and old, would have prognosticated his death. That some very serious injury was sustained by the brain, independent of the consecutive inflammation, will, we think, be allowed, both from the current symptoms, and the subsequent effects of the accident. But what was the nature of that injury? Would the application of the trephine over the seat of the external injury (we mean before reaction came on) have been of any service? we suppose this idea will be scouted by modern surgeons, who seem to have a *trepanophobia*—if we may be allowed such expression. The subjects of concussion and compression have afforded much scope for controversy—especially as regards their treatment; and it may not be uninteresting to those at a distance from the metropolis, to know the sentiments of one of our first surgeons—Sir Astley Cooper, on these matters. We have looked into the published lectures of Mr. Mingay Syders—and also into the still more recently published notes in the Lancet and Scalpel, from which sources the following observations are taken.

1. *Concussion.* Sir A. defines *concussion* to be simply the effect of a shock received by the brain, accompanied or not by organic lesion—while *compression* depends on either depression of bone, extravasation of blood, or formation of pus—any one or all of which will produce the same symptoms. He observes, that if a surgeon be called to a man in a state of stupefaction, not in a great degree, with regular pulse, easy breathing, the accident having existed some hours, the case is concussion, and not of a dangerous character. But if the patient has been first seized with vomiting, loss of muscular power, aberration of the mental faculties, intermitting pulse, unequal breathing,—then it is a dangerous concussion. In simple concussion, Sir A. observes, the patient when roused up, will have his pulse much accelerated—from 70, for example, to 120, or more. This, he thinks, is a diagnostic mark of concussion. The pulsation of the carotids is also greater in proportion than that of the other arteries of the body.

In the slighter cases of concussion, Sir Astley appears to think there is merely *a change in the circulation of the brain.* We should be more inclined to attribute the phenomena of concussion to the disturbance (occasioned by the shock) in the vital powers or properties of the cerebral mass itself, than to mere disturbance of the circulation. When concussion is violent, he observes, a "lesion of the brain takes place; but, when it is slight, no appearances can be discovered, on dissection, which indicate any alteration in structure." [Lancet.] This being the case, have we any proof that the disturbance of intellectual function is *entirely* dependent on change of the circulation?

In respect to the *treatment* of concussion, Sir Astley justly remarks, that the great danger we have to dread, is from inflammation. Blood-letting, therefore, is our sheet anchor. But it should not be rashly carried to too great an extent. Here, as elsewhere, we must be guided by the symptoms, which, to an attentive observer, will always indicate and contra-indicate blood-letting. Some degree of excitement or reaction in the brain, seems necessary after concussion, and this is to be controlled only, not entirely annihilated, by repeated and large bleedings. Sir Astley relates a case of concussion with slight laceration of the brain, which happened in one of the Borough hospitals, where the surgeon bled, and thought he could not bleed too much. He bled twice a-day, till the patient became pale, dejected, powerless, and, in ten days, lifeless, without manifesting any cerebral inflammation. On dissection, it was found that there was a slight laceration of the brain, with some extravasation of blood: but no attempt had been made by nature to heal the wound. Sir A. thinks, that the great abstraction of blood from the system prevented all attempt at adhesive inflammation in the brain. Still he admits that it is often necessary to take away blood, after the first large bleeding—but it must be in small quantities, watching the symptoms with the greatest possible care. He states the case of a gentleman who fell from his horse, while riding to London, and who received a concussion of the brain. He required the abstraction of 180 ounces of blood from the arm, besides leeching to the amount of 30 ounces more—and yet there was some hardness of the pulse, and inflammation of the brain after the very last bleeding. We need not dwell here on the highly dangerous and unscientific practice of bleeding the moment an accident of this kind occurs—merely because it has occurred. There is generally a recoil of the circulation, and a diminution of nervous energy for a time after the receipt of a serious injury—and to bleed in that period is preposterous. We should wait, of course, till the pulse rises, or other signs of reaction occur. We are then to use the lancet, to prevent inflammation, or extravasation, if there be rupture of vessels, or laceration of parts. In Mr. Syder's notes, Sir Astley is made to recommend local, rather than general bleeding—at least, to prefer temporal arteriotomy, or opening the jugular vein, to venesection by the arm. In the more recently taken notes, this practice is not insisted on. Small doses of calomel at night, with the sulphate of magnesia next morning, are recommended. Of emetics Sir Astley speaks with caution. He remarks, that nature's operations are generally salutary, and may afford us many hints. Vomiting is a very common occurrence after a severe injury—and Sir Astley avers that he has seen good effects from emetics in concussion of the brain. But he prudently dreads their effects where there is any extravasation of blood, or tendency to apoplexy. On this account, he always waits four or five hours after the accident before he orders them. His object appears to be, to "propel the blood towards the brain, and thus restore the powers of life." If the pathology of concussion be merely defective circulation through the vessels of the brain, then vomiting is well calculated to accelerate the circulation in them, and, indeed, in all the vessels of the body. But we fear our knowledge as to the exact condition of the brain in concussion is yet very limited, and not sufficient to ground a doctrine on. Then, again, can we certainly distinguish between concussion with, and without organic lesion?—we believe no man will pretend to this certainty—even after several hours have elapsed from the time of the accident. On all these accounts, we apprehend that emetics will be dangerous agents in the hands of the young, the inexperienced, or the inobservant practitioner. Where the stage of depression lasts long, and the supervention of reaction appears doubtful, we would be more inclined to give ammonia, and other grateful stimuli with caution, rather than hazard the violent commotion of an emetic. Where concussion of the brain, however, has taken place after much repletion or intoxication, which is often the case, then emetics are far less questionable than under opposite circumstances. As for the minor indications of treatment, they will readily present themselves to the mind.

As to trephining in cases of *concussion*, the operation cannot be defended on any other principle than the *suspicion of local extravasation* at the spot where the injury is received—and then the case becomes a compound of concussion and compression. We think, for example, that Mr. Cunningham's case, at the head of this paper, was a compound one of commotion and compression—else why should the permanent paralysis ensue. But in what part of the brain the compression existed, would have been difficult to ascertain. After reaction has come on, it would be only adding injury to injury, to apply the trephine.

2. *Compression.* Sir Astley lays down the main features or symptoms of compressed brain, as consisting of *stertorous breathing, slow pulse, and dilated pupils,* in addition to those of concussion already described. Compression results, of course, from extravasation of blood, depression of bone, or formation of matter. In the *first* case, the lecturer observes that the symptoms do not come on immediately. "The person first becomes stunned from the blow—after a little time becomes comatose, and it is difficult to rouse him—he is put to bed, and falls asleep—and in that sleep apoplectic stertor comes on." [Scalpel, p. 75.] The seat of the extravasation may be between the meninges, or into the substance of the brain—but this makes no difference in the symptoms, excepting that if a coagulum of blood presses on the origin of a particular nerve, there will be partial paralysis of the part supplied by that nerve. *Treatment.* If the extravasation be accompanied by fracture, the trephine may be applied with advantage, before any excitement takes place; the bowels are to be kept open, and the patient quiet. In addition to these, we must deplete freely, to prevent inflammation. But if there be no bruise or fracture to indicate the situation of the extravasation, it would be madness to seek for it with the trephine. Even when the trephine is applied, we can only remove blood that is extravasated outside of the dura mater. We are not authorized to cut through that membrane, to liberate any fluid beneath.

Fracture of the skull, Sir Astley properly remarks, is not in itself dangerous, unless accompanied by depression, extravasation, or concussion. The object is to prevent inflammation. For mere fracture, no operation is now, of course, ever thought of. We quote the following passage from page 78 of the Scalpel.

"In fracture, with an extensive opening, approximate the edges of the wound immediately, and heal it as quickly as possible; as you thereby prevent inflammation of the dura mater, which is very apt to occur in these cases. If there be concussion, or compression, with fracture, you may trephine, but do not be in a hurry; as, when I was a dresser, I have often sent for a surgeon, to a patient labouring under compression or concussion; yet, by taking away blood in the interim, the symptoms of concussion or compression have disappeared. Delay then the operation, till you have tried for some time to relieve the symptoms by depletion, by bleeding, and purging. For the symptoms of concussion, the treatment must be such as I described in my last lecture. If the fracture should be accompanied with extravasation, deplete; and if that should not succeed, trepan." 78.

*Depression.* Sir Astley observes that there is some difference of opinion on the practice in these cases. When fracture is accompanied by depression, the symptoms come on immediately, as the cause is immediate. Sir A. tried an experiment on a dog, whose head he trepanned for this purpose. Having detached the dura mater from the bone, for some distance around the perforation,* the experimenter compressed the brain slightly with his finger, without any perceptible effect. On increasing the pressure, the animal shrunk, and endeavoured to escape. The pressure being still further increased, coma, loss of power, slow and irregular pulse were produced. The pressure removed, the animal soon recovered his power, though apparently giddy, and scampered off. Sir Astley mentions a source of deception respecting depression, which we have many times witnessed. A man, and particularly a young person, gets a severe blow on the *head*, by which the integuments are puffed up into a tumour. In the centre of this tumour there will be a feeling as if the bone was considerably depressed or beaten in, and it is not easy to persuade an inexperienced surgeon to the contrary. Yet, in five or six days, all will come smooth again, and the bone will be found entire. The external table of the skull, too, will often be beaten in, in adults, (for in old and young people there is no diplœ) without any injury to the internal table.

"I divide fractures of the skull into *simple* and *compound*, as the treatment differs in the two cases very considerably; there is also concussion, or compression, or not. Let us take, then, this case: suppose you are called to a patient, who has a portion of bone driven in, yet, on speaking to him, you find his mind perfect; here, then, are no symptoms of concussion or compression. But, on the contrary, suppose you are called to a case, where the person has lost the powers of mind and body; here you have concussion, or compression, or both. If fracture be *simple*, viz. if there be no wound, and no symptoms of injury to the brain, it would be the worst of practice to make an incision through the scalp, and trephine; for it would add to the danger,

* See our remarks on Dr. Kellie's paper, in another part of this number, respecting pressure on the brain from trepanning.—Rev.

by making that a compound fracture which was before only a simple one; for inflammation rarely follows when the fracture is simple, but often after an incision has been made; therefore, if you can, always avoid it, never make an incision through the scalp, where there is fracture, if there be no symptoms of injury of the brain; if there be symptoms of injury to the brain, draw blood, purge, and see how far they are the symptoms of concussion; and then, and not till then, trephine. A great many years ago, I was called in by a surgeon, to a lady, who had fallen against the fire-place, and a piece of the bone of the forehead became depressed; there were no symptoms of injury to the brain, and she recovered by depletion alone. The practice, formerly, was to make an incision in almost every case. When inflammation of the dura mater, or the membranes of the brain, has once existed, you do not retard it by trephining, and the patient goes gradually to death. I will give you two, of many, instances, on which my opinions are founded; one occurred to Mr. Cline, and the other to Mr. Birch. A man came from Walworth, and was under the care of Mr. Cline; he had received a blow on the head, which had forced the bone in. Mr. Cline said to the patient, "here is a case of depression, you had better submit to the operation of trephining;" the man said "I am no judge, do what you please, Sir;" the man had no symptoms of injury of the brain, and walked into the operating theatre; the operation was performed, and he walked out again; no bad symptoms came on, and he soon got completely well. The patient, with depression, under the care of Mr. Birch, would not have the depressed portion removed; the consequence was that, in eleven days, pain of the head came on, and inflammation, when he lost the powers of his mind, and was operated upon, but it did not at all arrest the symptoms, and he died of inflammation of the brain. In the other hospital, two boys were admitted; one for depression of a portion of the os frontis, from the kick of a horse; the other for the same accident from a blow; one had the portion of bone raised, and did very well; in the other, the operation was delayed, in consequence of the interference of his mother, and the boy died of inflammation, after having been trephined. Inflammation does not always follow fracture with depression. If there be fracture with depression, and that exposed, I use the elevator; put it under the depressed portion, and raise it; if the bone be comminuted, you can remove the loose portions, and raise the depressed part; but, if it be wedged, I would trephine. I have found that, from raising the depressed portion of bone, no mischief ensues; but if you do not raise it, inflammation will arise. Two circumstances I will call to your attention; when fracture occurs with depression, a small spicula of bone will sometimes enter the brain, and produce epileptic fits. I remember this to have occurred in a negro, who had a depression of bone, and epileptic fits, which continued for a year. When the portion of depressed bone was removed, and when the trephine had sawed through the bone, the surgeon tried to raise it, but could not; at last, with some difficulty, it was detached, and on examining it, a thorn of bone was found attached to it, which had penetrated the brain, exciting epileptic fits. He had only one fit after, and completely recovered. But the next case is the most extraordinary that I am acquainted with, and I am surprized it has not had a greater effect on the public, in a medical and physiological point of view. A man was pressed into His Majesty's service, early in the beginning of the late revolutionary war. He was taken to the Mediterranean, and there received a fall, I believe from the yard-arm; he was picked up on the deck, insensible. The vessel soon after made Gibralter, and he was put into the hospital there, where he remained some months, insensible; he was then brought on board the Dolphin frigate, to Deptford; the surgeon who attended him there, was one day visited by Mr. Davy, a dresser of this hospital. The surgeon said to Mr. Davy, "I have a curious case of a man who has been insensible for a long time; his breathing is rather laborious, his pulse natural, and it corresponds with the working of his fingers; but he lies on his back, deprived of volition and sensation." Mr. Davy accompanied the surgeon to see him, and he found that there was a slight depression of the head. Mr. Davy said, "send him to St. Thomas's Hospital;" he came, and was under the care of Mr. Cline. He was found lying on his back, breathing with considerable difficulty, with regular pulse, and each time the pulse beat, the fingers moved, so that you might tell his pulse by his fingers—If he wanted food, he moved his lips or tongue; that was the sign. Mr. Cline found a depression, and operated upon him. *Thirteen months* and a few days after the accident, he was operated on by the trephine; and the depressed portion of bone was elevated.—Whilst laying on the table, so soon as the portion of bone was raised, the fingers immediately ceased working. The operation was performed at one; and at four in the afternoon, I was going round the wards, and saw this man raised on his pillow; I went up to him, and said, "have you any pain?" he put his hand to his head: volition and sensation had returned, and in four days, he got out of bed, and conversed; in a few days more, he told us where he came from, of his being pressed, of his being carried down to Plymouth, or Falmouth; but from the moment of the accident, thirteen months and a few days, oblivion had come over him; all recollection had ceased; he had, for that time, drank of the cup of Lethe; and there had been cessation of almost all the bodily and mental functions; yet, on removing a small piece of bone, he was restored to his powers of mind and body. Thus, you will see, that you must not be deterred from performing the operation by *any length of time*, for still you may be able to restore the powers of the mind and the body."*

We shall occasionally introduce the opinions of this celebrated surgeon and teacher, when surgical points are under discussion, for the benefit of those at a distance from the metropolis. Sir Astley has permitted his lectures to become public property through three, if not more,

* Scalpel, p. 79—80—81.

separate channels, and, therefore, there is no longer any delicacy necessary in quoting them *pro bono publico*.

---

2. *Extirpation of the Thyroid Gland.* A terrible operation of this kind was lately practised by Dr. Graef, of Berlin. The patient was twenty-two years of age, and the thyroid tumour had been growing from the age of fourteen. It was now an enormous size—as large as a child's head! They first tried ligature of the superior thyroid artery; but this was followed by alarming symptoms, as insupportable head-ache, and violent pains in the neck, and in the tumour itself. The patient loudly demanded extirpation of the gland, and Mr. Graef consented to the enterprize. We cannot detail the steps of the operation, but it will convey some idea of the difficulty experienced, when we state that it was necessary to take up forty-three arteries, and that the tumour, when removed, weighed two pounds and a half!—The operation lasted half an hour, and was followed by complete success. —*Heden on the Thyroid Gland.*

---

3. *Dr. Civiale's Lithontriptor.* It was but lately that we presented our readers with the experiments and proposals of Messrs. Prevost and Dumas respecting the dissolution of calculi in the bladder by means of the galvanic fluid. Since that period, a still bolder flight has been attempted, and the long-imagined, but never effected, operation of breaking down calculi in the bladder by means of an instrument, has, it is said, at length taken place. Messrs. Chaussier and Percy have been deputed to examine into the merits of this operation, and have reported to the Royal Academy of Sciences accordingly. Several people have already put in their claims for participation with Dr. Civiale in his invention, and some even claim priority. It is stated, indeed, by M. Percy, that the project was announced in the Saltsburgh Medical Gazette, so far back as 1813, by Dr. Gruithuisen. It does not appear, however, that the German ever put his proposal into execution, and therefore the more substantial merit of actual reduction to practice belongs to Dr. Civiale.

The first principle or condition of the operation consists in dilatation of the urethra, and the introduction of a *straight sound* into the bladder. This last is indispensible. Through the straight and hollow sound is introduced another, containing a steel apparatus consisting of three elastic and curved branches, to seize and fix the stone when projected. This, of course, is an old invention. Within this last apparatus is a steel stilet, at the extremity of which is a circular saw, which can be worked upon the stone, till it is destroyed by repeated drillings. We shall give a short description, however, in the original, as it is not easy to reduce the French technicals into English.

"C'est encore une sonde, mais une sonde d'acier pouvant entrer dans la première, droite et creuse comme elle, et portant trois branches très-élastiques, courbes, restant rapprochées et invisibles tant qu'elles sont enfoncées dans la sonde principale, qui leur sert gaîne, et quand on les pousse au dehors, s'épanouissant par l'effet de leur ressort, et formant comme une cage, une bourse d'acier où l'on parvient plus ou moins vite à faire entrer la pierre, sur laquelle on la ferme aussitôt en retirant la sonde à soi, c'est-a-dire en arrière, autant que le volume du corps étranger, ou le sens dans lequel il a été chargé, peuvent le permettre.

"Dans la seconde sonde, ou plutôt dans le cylindre formant la pince, est un long stylet d'acier, qui y entre et peut y tourner librement, et qui se termine, du côté de la vessie, et entre les serres de la pince, par une lime en fraise, ou par une petite scie circulaire, un trépan pyramidal, un simple carlet, selon la circonstance, la grosseur et la nature présumée de la pierre. Celle ci étant bien fixée, on pousse contre elle le stylet mobile, et au moyen d'une poulie dont il est pourvu à son extrémité extérieure, d'un tous d'horloger sur lequel on le monte, et d'un long archet à corde de boyau, on le fait tourner comme quand on veut percer un trou dans une plaque de métal. A peine la machine est en activité, qu'on entend le bruit sourd, ou sonore du broiement ou du brisement qui s'opère sur le calcul, selon la mollesse ou la dureté dont il jouit, et le patient ne manifeste que très-peu ou point de douleur.

"A mesure que le travail avance, on fait marcher dans la même proportion le stylet contre la pierre, en suspendant un moment l'action de l'archet, que l'on reprend bientôt, pour comminuer de plus en plus la concrétion ennemie, et hâter, si l'opérateur ou le malade ne sont pas trop fatigués, l'œuvre de sa destruction, laquelle, ne devant s'achever qu'à deux ou trois reprises, est ajournée à des termes plus ou moins rapprochés. Une miction spontanée ou une injection d'eau tiède dans la vessie termine ordinairement la séance, et fait rejeter par l'urètre, qu'a dilaté la grosse sonde, des éclats, des fragmens plus ou moins nombreux et considérables, ou du sédiment borbeux qui se précipite bientôt et qu'on peut recueillir aisément."

The operation has been performed many times on the dead, and sometimes on the living body. The bladder is secure from the effects of the terebration, and it is asserted that no danger can arise from the operation. After having witnessed three operations on the living body, Messrs. Chaussier and Percy conclude thus:—"after all that has passed —and wishing to steer a middle course between enthusiasm which exaggerates, and distrust which repels every invention, we are of opinion that the new method proposed by Dr. Civiale for destroying a stone in the bladder is equally glorious for French surgery, honorable to its inventor, and consolatory to humanity;—and that, notwithstanding its inefficacy in some cases, and the difficulty of its application in others, it cannot fail to form an epoch in the annals of the healing art, and be regarded as one of its most ingenious and precious resources."

It would be wrong in us to hazard any prognostication on an operation which has been actually performed several times on the living body, before such competent judges as Chaussier and Percy; but yet we can not help fearing that the difficulty of introducing a *straight sound* large enough to contain efficient pincers to secure, and borers to penetrate a stone of any magnitude in the bladder, will be such as shall prevent its ever coming into general use. Time, however, will tell—and most sincerely do we hope that we may turn out to be false prophets upon this occasion. We will, however, hazard a conjecture that the forceps for extracting *small stones*, suggested by Sir Astley Cooper, and constructed by Mr. Weiss, will prove of far more general application than any instrument for the destruction of stones of magnitude in the human bladder—unless such instrument be introduced through an opening into the urethra made *in perineo.* In this last case, we think it probable that an apparatus of considerable power might be passed into the bladder, and made to act on a calculus there.

P. S. It is curious, that a general who was operated on for stone, a few years ago, planned out to the writer of this article, an apparatus almost identically the same as that constructed by Dr. Civiale; but he was not aware that a straight sound or instrument could be introduced into the bladder.

---

4. *Stricture of the Urethra.* Mr. Arnott [Med. Chir. Trans: vol. xii] has lately called the attention of the surgical profession to the treatment of old and narrow stricture, by incision, in preference to destruction of the parts by caustic, or forcing the obstruction by mechanical means. He relates a case in elucidation.

The patient (forty-nine years of age) had suffered from stricture of the urethra for fifteen years, and, during the last four, had not been able to get a bougie into the bladder. When he came to M. Arnott, he was found to make water every hour, attended with much straining and pain; the urine coming only *guttatim.* A bougie was stopped towards the bulb of the urethra, and could not be got further, notwithstanding several trials. The caustic was next applied, but without benefit. In consultation with Mr. Shaw, an operation was determined on. A catheter being introduced down to the obstruction, a free external incision was made upon its point, and an opening made into the urethra, anterior to the obstruction. The point of a very small grooved probe was then guided into the aperture, and pushed on towards the stricture, into which it entered with little difficulty, and went on to the bladder. Upon this a bistoury was run down, and the strictured portion divided, occupying about a quarter of an inch in extent. The catheter was now carried onwards, with great facility into the bladder, and upwards of a pint of urine was drawn off. No unfavourable symptom occurred. On the fourh day the catheter was withdrawn (as urine passed between it and the urethra) and it was replaced by a larger sized one of elastic gum. This was again replaced by one of silver, still larger. The wound healed favourably, and was quite closed by the end of a fortnight. The catheter was then withdrawn during the day, the patient now being able to make water in a full stream. The instrument, however, was worn at night, for another week, when it was left off, and a bougie introduced occasionally.

For Mr. Arnott's observations on the advantage of such an operation over caustic, or what is called forcing the stricture, we must refer to the paper itself. His observations appear reasonable.

---

5. *Ectropopharynx.* This is a hard name for an instrument, to be used in a hazardous operation—œsophagotomy. When foreign bodies become lodged in the œsophagus, and we cannot extract them by the mouth, or push them down into the stomach, it is evident that there is no other resource than cutting into the œsophagus, for the extrication of the obstructing body. M. Vacca-Berlinghieri, of Pisa, proposes an instrument resembling a catheter, but rather stronger, to which he gives the above name, and which, being introduced down to the obstruction, is to be pressed outwards, by pushing the handle in an opposite direction, as we push the staff to one side in the operation of lythotomy. By this process, he thinks, we may cut down on the projecting œsophagus with much less danger of wounding nerves or blood vessels.

For our own parts, we do not see what greater advantage or facility there is in cutting down upon a catheter in the gullet, than upon the obstructing body itself. The *latter* must always form a projecting point and guide for the knife. Besides—in operating according to Berlinghieri's plan, the opening into the œsophagus must necessarily be *above* the obstruction—whereas, in the common way, we cut down upon the very spot where the foreign body is lodged.

---

6. *Stricture of the Urethra.** Mr. Shaw does not attempt to embrace, in this paper, all the effects of stricture, but confines himself to the following four pathological facts.

"1st.—I have not," says he, "in more than a hundred dissections which I have made of diseases of the urethra, seen a stricture or narrowing of the canal, posterior to the ligament of the bulb; nor have I been able to find one example of stricture beyond this part among those preserved in the College Museum.

"2nd—In almost every instance where a narrow stricture has existed for some time, in any part of the urethra anterior to the ligament of the bulb, I have found the membranous and prostatic portions dilated to three or four times their natural size.

"3rd—The ducts of the prostate, which are naturally very small, are always more or less enlarged when there has been a stricture or long continued irritation of the canal.

"4th—When such a stricture as causes occasional retention of urine has existed for some years, the bladder is found to be not only thickened, but often at the same time sacculated." 463.

---

* On the Effects of Stricture of the Urethra, particularly of the sacculated state of the bladder, with an enquiry into a mode of treatment to avert this latter consequence of stricture, which is often fatal. By John Shaw, Esq. [*Med. Chir. Trans. Vol.* xii.]

If the first three observations be correct, some practical rules may be deduced from them. Thus, if in passing down an intstrument, we meet with obstruction *posterior* to the ligament of the bulb, we ought not to impute it to stricture of the passage, but to some other cause, not removable by the means employed for stricture. In respect to the second observation, that the membranous and prostatic parts of the urethra become enlarged in consequence of stricture; if such be the case, it will be admitted that obstruction to the bougie posterior to the bulb, in a patient who has previously had stricture anterior to it, will render it improbable that such obstruction results from narrowness or stricture of the canal. The rule to be deduced from these observations is, that on feeling an obstruction posterior to the ligament of the bulb, we should not persevere in the attempt to push the instrument further in. It is probable, in such cases, that the point of the instrument is entangled in one of the dilated ducts, in which case, any attempt to force the catheter into the bladder may probably form a false passage. If the catheter enters into one of the enlarged ducts, Mr. Shaw observes, it may be pushed through the prostate into the back part of the dilated bladder, several preparations of which accident are in the collection in Windmill Street. These observations, he thinks, are necessary when, even in the present day, the authority of Dessault is given for using forcible means to overcome obstructions on the prostate part of the urethra.

Our author next adverts to the anatomy of the parts, and shews that from the sudden narrowing of the canal at the bulb—and the curve which it naturally takes there, the mechanical impediments to the introduction of an instrument, at this point, are greater than at any other.

"If to these impediments we add the difficulty occasioned in the living body by the contraction of the muscles which surround this part of the urethra, and which is always excited by a slight inflammation of the membrane, we shall understand how the spasmodic affection, which comes on the moment a bougie touches the inflamed part, combined with what I have called the mechanical difficulties, may produce so complete an obstruction to the entry of an instrument, as to give rise to the idea of the presence of stricture." 467.

Error may be increased on these occasions, by seeing a cut or indentation on the bougie, caused by pressure against the lower edge of the ligament exactly resembling that which is considered as unequivocal proof of stricture. So much delusion has indeed prevailed respecting the existence of stricture, that we are not much surprized at the histories which are published of sudden cures of narrow strictures at the bulb, by bleeding, antispasmodics, &c. It is also not improbable, as Mr. Shaw observes, that actual strictures at the bulb have originated in the inflammation consequent upon the ineffectual attempts to pass an instrument through this part of the urethra, while its lining membrane was in a state of irritation.

In respect to the sacculated state of the bladder, it is observed by all who are in the habit of examining the urinary organs after death, that this condition is a very common occurrence where stricture has existed. Although our author is unable to point out any particular diagnostic symptom, by which the sacculated state of the bladder can be known during life, yet he ventures "the opinion that, when in severe cases of stricture there is a peculiar irritation about the back part of the bladder, and between it and the rectum, especially after voiding urine, we may suspect that a sac has formed."

The questions then occur—is such a sac ever spontaneously removed? Will not a quantity of urine lodge there?—and what will be the consequence of such lodgment?—The *first* question, Mr. Shaw thinks, can never be answered with certainty—the *second*, he fears must be answered in the affirmative—and, as to the *third*, and most important, he would be inclined to say, that "the lodgment of urine in a sac produces a very peculiar train of symptoms, constituting a disease that is often fatal, the patient's death being occasionally preceded by symptoms of peritonitis." Mr. Shaw has also observed that the sufferings of the patient are of a nature very different from what are considered the more common consequences of stricture.

There is reason also to fear that these lodgments of urine in sacs of the bladder may cause the formation of calculi there. Another consequence, scarcely alluded to by authors, is a fistulous communication between the rectum and bladder, sometimes resulting from the formation of a sac in the bladder—more usually from a sacculated state of the prostate. Both of these have their origin, generally, in stricture. The important fact, too, must not be overlooked, that the prostate itself is very liable to become sacculated, even without the presence of stricture. The next object of enquiry is, can we avert the abovementioned consequences of obstructed urethra?

Suppose a patient has had stricture near the bulb for several years, which has resisted every plan of treatment, and now will not permit the passage of the smallest bougie. The patient has frequent attacks of inflammation of the bladder, and the water dribbles away slowly, or is passed guttatim. The usual means, we say, having failed, what can we hope for but a suppuration in the perineum, and a fistulous opening for the water, as the most favourable issue, bad as it is? In the state above described, it is evident that (while the obstruction continues) the patient is daily liable to complete retention of urine, from catching cold, or any irregularity.

"If this should happen, what must be the consequence? The state of the stricture is such, that neither a catheter nor a bougie can be passed; therefore, if the patient be not immediately relieved, and this with great care, by cutting into the perineum, or by puncturing the bladder, he must either die of the irritation caused by the distended bladder, or the urethra will burst behind the stricture, and the urine necessarily in a highly acrid state, be effused into the scrotum. If this last should be the result (which it too commonly is in such cases) and if the patient be not then treated with skill and decision, he will probably die in the course of three days; or, should he escape the immediate danger, he will run much hazard of sinking under the extensive sloughing of the scrotum and penis, which almost invariably follows rupture of the urethra, when a free passage for the evacuation of the effused urine has not been made." 473.

Even if things do not go the above length, from complete retention, yet the constant state of irritation in which the bladder is kept, will either lead to irritative fever of a dangerous and wasting kind, or induce a disorganized or sacculated state of the prostate gland, or of the bladder itself, under which the patient will ultimately sink. What is to be done, Mr. Shaw asks? Should a catheter be forced through the stricture, or should the bladder be punctured? The first is decidedly wrong, he avers, because the contracted portion of urethra is probably much firmer and stronger than any other part of the canal. Puncturing the bladder would be infinitely safer, but it would give only temporary relief.

Mr. Shaw proposes an operation, then, which is not severe, nor attended with danger, if properly performed—which will not only give temporary relief, but also put the patient into a condition of much greater ease and comfort than could be expected. The operation "*is merely to cut through the stricture, to introduce a catheter from the glans, and endeavovr to make the urethra entire—by allowing the wound to granulate over the catheter.*" It is to be remembered that this operation is not proposed for the spur of the moment, when the patient is in danger from retention of urine. It is to be performed before things come this length, and before danger is so imminent. The only difficulty, Mr. Shaw remarks, likely to occur in the first stage of such an operation is, the chance of the point of the catheter getting into one of the false passages, when passing it down to the stricture, as a mark for our incision into the urethra. In the second stage of the operation, we may have some difficulty in discovering the opening of the urethra, after the stricture is cut through, for there may be false passages continued even beyond the point of stricture—or the urethra, by its elasticity, may be so close that we cannot see it. These difficulties have occurred in one or two cases, but are surmounted by observing the point from which the urine issued. For this purpose the urine ought to be retained for some time previously to the operation. If this cannot be done, we are to desist from prosecuting the operation till the bladder again fills, when we are to narrowly watch the issue of the stream, and pass a catheter through the opening into the bladder. It is scarcely necessary to add that, after the wound has healed over the catheter, the urethra must, for a considerable time, be kept free by the use of the bougie. The worst evil that could befal this operation would be the non-closure of the wound—in other words, a fistulous opening in perineo. But even *this* is the best accident that could befal the patient, if the operation were not performed. Again, the fistula, after operation, would differ materially from a spontaneous one. Thus the stricture would be removed, which is the first step towards the cure of fistula, and there would be only a simple wound in place of the multitude of callous sinuses which take place in spontaneous fistula. Our author concludes by recommending the early performance of such an operation, before the parts get disorganized and hardened. He has lately seen a patient on whom this operation was performed, and his appearance was so much altered for the better, that he scarcely recognized him. Previous to the operation he was so reduced by constant irritation, that he appeared like a broken down man of sixty—but now he is a hale and strong looking man.

We think Mr. Shaw's paper is entitled to the serious attention of his surgical brethren, and himself to the thanks of the profession.

